# Evaluation of Bone Marrow Texture and Trabecular Changes With Quantitative DCE-MRI and QCT in Alloxan-Induced Diabetic Rabbit Models

**DOI:** 10.3389/fendo.2021.785604

**Published:** 2021-12-22

**Authors:** Pianpian Chen, Yunfei Zha, Li Wang, Liang Li, Lei Hu, Dong Xing, Baiyu Liu, Liu Yang, Qi Yang, Changsheng Liu, Huan Liu, Weiyin Liu

**Affiliations:** ^1^ Department Radiology, Renmin Hospital of Wuhan University, Wuhan, China; ^2^ Precision Healthcare Institute, GE Healthcare, Shanghai, China; ^3^ MR Research, GE Healthcare, Shanghai, China

**Keywords:** Diabetes MILES Study, lumbar marrow, DCE-MRI, texture analysis, quantitative CT

## Abstract

**Purpose:**

To investigate whether the microvascular permeability of lumbar marrow and bone trabecular changes in early-stage diabetic rabbits can be quantitatively evaluated using dynamic contrast-enhanced magnetic resonance imaging (DCE-MRI), quantitative computed tomography, and texture-analyzed permeability parameter *K^trans^* map of DCE-MRI.

**Materials and Methods:**

This prospective study included 24 rabbits that were randomly assigned to diabetic (n = 14) and control (n = 10) groups. All rabbits underwent sagittal MRI of the lumbar region at 0, 4, 8, 12, and 16 weeks after alloxan injection. Pearson correlation coefficient was performed to determine the correlation between permeability parameter and bone mineral density (BMD). Repeated-measures ANOVA was used to analyze the changes in lumbar BMD over time in each group and the texture parameters of diabetic rabbit lumbar marrow at different time points. Mann–Whitney U rank sum test was used to compare the differences of each index between the two groups and calculate the area under the curve (AUC).

**Results:**

BMD was correlated with *K^trans^*, *K_ep_*, and *V_e_* but not with *V_p_*. At weeks 0–16, the BMD of the rabbits in the diabetic and normal groups was not statistically significant, but the change in BMD showed an overall downward trend. For texture analysis, entropy, energy, and Uniformized positive pixel (UPP) parameters extracted from the *K^trans^* map showed significant differences from week 0 to 16 between the two groups. The identification ability at 8–12 weeks was higher than that at 12–16 weeks, and the AUCs were 0.734, 0.766, and 0.734, respectively (P < 0.05 for all).

**Conclusions:**

The changes in BMD measured using quantitative computed tomography occurred later than those measured using bone trabecular morphometry. Texture analysis parameters based on DCE-MRI quantitative parameter Ktrans map are feasible to identify early changes in lumbar marrow structure in diabetic rabbits.

## Introduction

The global prevalence of type 1 diabetes mellitus has been increasing by 2%–5% annually, leading to elevated occurrence of diabetes-related pathologies, such as bone disorders (1), cartilage alterations, and bone loss (2, 3). The main complications of diabetes, osteopenia, and bone microstructural changes increase the possibilities of fractures and friability and decrease bone strength (4). A meta-analysis of 16 studies reported no difference in lumbar spine bone marrow density (BMD) between adults with type I diabetes (T1D) and healthy subjects, controlling for age and gender effect (5). The associations of glycemic control and diabetes duration with BMD reported in many studies have been contradictory, but microvascular complications (retinopathy, neuropathy, and nephropathy) have been found in low-BMD patients with T1D (6–8). The pathophysiological mechanism of diabetic bone diseases remains unclear, but little evidence indicates that diabetic bone disease might be a chronic microvascular complication (9). In addition, the relatively modest BMD reduction in patients with T1D does not fully account for increased fracture risk.

Due to the high transport rate of MRI contrast agents such as paramagnetic Gd(III) –chelate (10), quantitative dynamic contrast-enhanced magnetic resonance imaging (DCE-MRI) based on a pharmacokinetic model can reflect the alterations of bone marrow permeability in early diabetes stage (11). Radiomics has been used to predict treatment outcomes and time-dependent reactions based on so-called quantitative texture features (i.e., the distribution patterns of pixelwise signal intensities of given images). Zhang et al. (12) reported here that an Fe_3_O_4_ nanoparticle-based glutathione (GSH)-responsive MRI probe correlate between the interlocked MRI signals and local GSH concentration was established and further applied for mapping the heterogeneous distribution of GSH within an intracranial tumor *in vivo*, which will offer a practical route for quantitatively mapping tumor-specific biomarkers *in vivo*. Application of texture analysis on medical images, such as MRI, CT, and plain radiographic images, has demonstrated great potential in disease diagnosis (13) and lesion detection and characterization (14) and even in monitoring disease progression and longitudinal evaluation of emerging therapies (15). However, no radiomic report on diabetic permeability maps, which are likely to detect early diabetes-induced abnormal bone alterations, is available. Therefore, investigating the potential role of texture analysis based on permeability maps and determining texture analysis results may provide more information on the relationship between lumbar bone marrow heterogeneity and permeability occurrence.

Current studies have indicated that quantitative computed tomography (QCT) can more sensitively detect the changes in cancellous bone microstructure caused by changes in bone turnover (16). Since QCT can measure bone mineral content per unit volume of cortical bone and cancellous bone (13), it has unique advantages in evaluating diabetic lumbar vertebra disease and could act as a reference of true volumetric BMD to reflect the spinal structure stability. Determining the feasibility of measuring bone density using QCT combined with texture analysis on MR permeability map is important to investigate the complex relationship between diabetic bone marrow microvascular disease and bone density, bone structure, bone transformation, and fracture risk assessment.

The aim of this study was to evaluate the correlation between bone marrow microvascular permeability parameters, BMD, and quantitative trabecular bone morphometric parameters with DCE-MR and QCT in alloxan-induced rabbit diabetic model. We also investigated whether texture analysis based on the permeability parameter map could evaluate the microstructural changes in lumbar trabeculae in early-stage diabetic rabbits.

## Materials and Methods

### Animals and Diabetic Rabbit Model

This experiment was reviewed and approved by the ethics committee of our university. Twenty-four healthy adult male Japanese white rabbits (fasting weight, 2.8–3.1 kg; average fasting weight, 3.0 ± 0.1 kg) were provided by our Animal Experimental Center. All rabbits were kept for 1 week. They were fasted for 12 h before being modeled and allowed to eat and drink water within 48 h afterward. The fasting blood glucose level was confirmed to be <6.0 mmol/l with an average of 5.5 ± 0.3 mmol/l. The rabbits were randomly divided into diabetes (n = 14) and control (n = 10) groups. For the diabetic group, alloxan monohydrate (100 mg/kg of body weight; Sigma-Aldrich Chemical, St. Louis, MO, USA) was dissolved in sterile normal saline to achieve a 5% concentration and was immediately administered intravenously *via* the marginal ear vein over a 2-min period by using a 25-gauge butterfly catheter. The diabetic state was achieved 48 h later and was verified by quantitative determination of blood glucose levels. The control rabbits were injected with 100 ml/kg physiological saline. After 48 h, the peripheral blood glucose concentration was measured with a blood glucose meter (Sannuo blood glucose meter). When a single measurement showed a peripheral blood glucose level ≥14 mmol/l or two measurements showed a peripheral blood glucose level ≥11 mmol/l, the rabbits were identified as being successfully modelled (17). Blood glucose levels were monitored in all rabbits in both groups weekly by using the Optium Xceed glucometer (Abbott, Bedford, MA, USA) for the subsequent 4 weeks and tended to stabilize after 4 weeks. Subsequently, the blood glucose level was measured every 4 weeks before the MRI and CT examinations.

### Routine MRI and DCE-MRI Examination

All MRI scans were conducted with a 3.0 T MRI scanner (Discovery MR750; GE Healthcare) at 48 h as baseline and at the fourth, eighth, 12th, and 16th week after the successful modeling. These rabbits were anesthetized with an intravenous injection of 3% sodium pentobarbital solution (1.3 ml/kg) through the ear margin. After successful anesthesia, all rabbits were fixed in the supine position into an eight-channel phased-array knee coil. The sagittal fast spin echo T_1_-weighted imaging (FSE-T_1_WI) (TR = 300 ms, TE = Min Full, slice thickness = 3 mm without gap, 12 slices were acquired, FOV = 160 mm × 160 mm, in-plane matrix = 320 × 288), FSE-T_2_WI (TR = 2,500 ms, TE = 120 ms, slice thickness = 3 mm without gap, 12 slices were acquired, FOV = 160 mm × 160 mm, matrix = 320 × 2 88), and DCE-MRI based on liver acquisition volume acceleration sequence (before contrast injection: flip angles of 9° and 12° before contrast injection and of 10° after contrast injection, TR = 3.5 ms, TE = 1.6 ms, slice thickness = 3 mm, 12 slices, FOV = 200 mm × 160 mm, matrix = 192 × 192) of lumbar vertebrae were scanned. Before contrast injection, two liver acquisition volume acceleration sequence scans were used to obtain T_1_ mapping. The acquisition time was 8 s for a single three-dimensional (3D) volume, and 35 continuous temporal phases were acquired. A dosage of Omniscan (Gadodiamide, GE Healthcare, Ireland) of 0.2 mmol/kg was injected at a flow rate of 1.0 ml/s. After two dynamic phases were scanned as baseline, the contrast agent was injected into the rabbit ear vein by using a US MeoRao double-tube high-pressure syringe, followed by 5-ml physiological saline.

### QCT Examination

After anesthesia, the rabbits were placed in the supine position and the lower limbs were straightened. A solid phantom (V.4.0) was placed under the waist, and the lumbar vertebrae of the rabbits were scanned with GE Bright Speed 16-slice spiral CT. The scanning line was parallel to the upper and lower edges of the experimental rabbit lumbar vertebral body. The scanning parameters were as follows: tube voltage = 120 kV, tube current = 250 mAs, bed height = 179 cm, screw pitch = 0.531, SFOV = large body, matrix = 512 × 512, slice thickness = 1 mm, standard algorithm reconstruction.

### Data Measurement and Analysis

**Figure 1:**
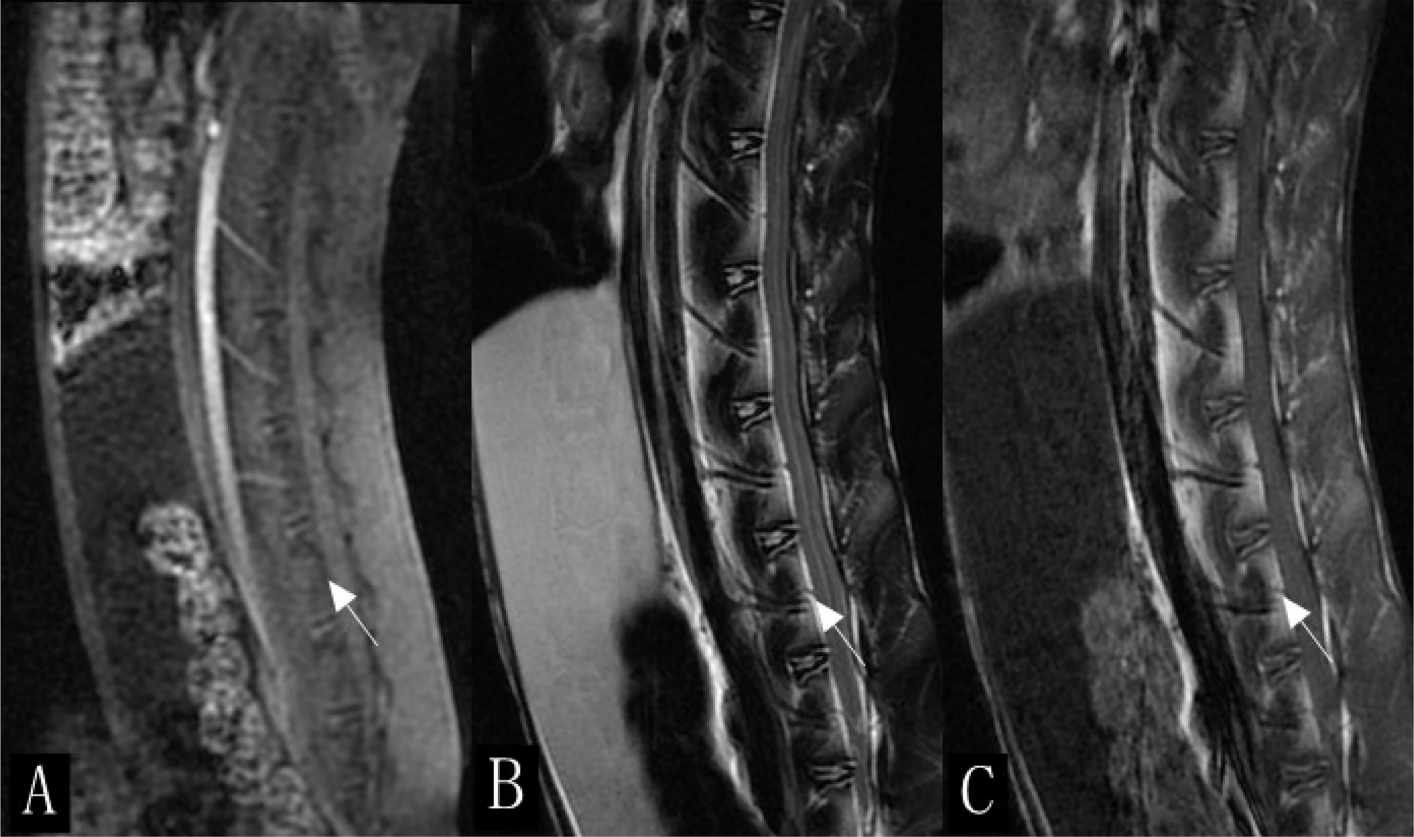
Sagittal scan of lumbar spine in rabbits (white arrow). **(A)** Enhanced scan image. **(B)** T2-weighted imaging (T_2_WI). **(C)** T_1_WI.

**Figure 2:**
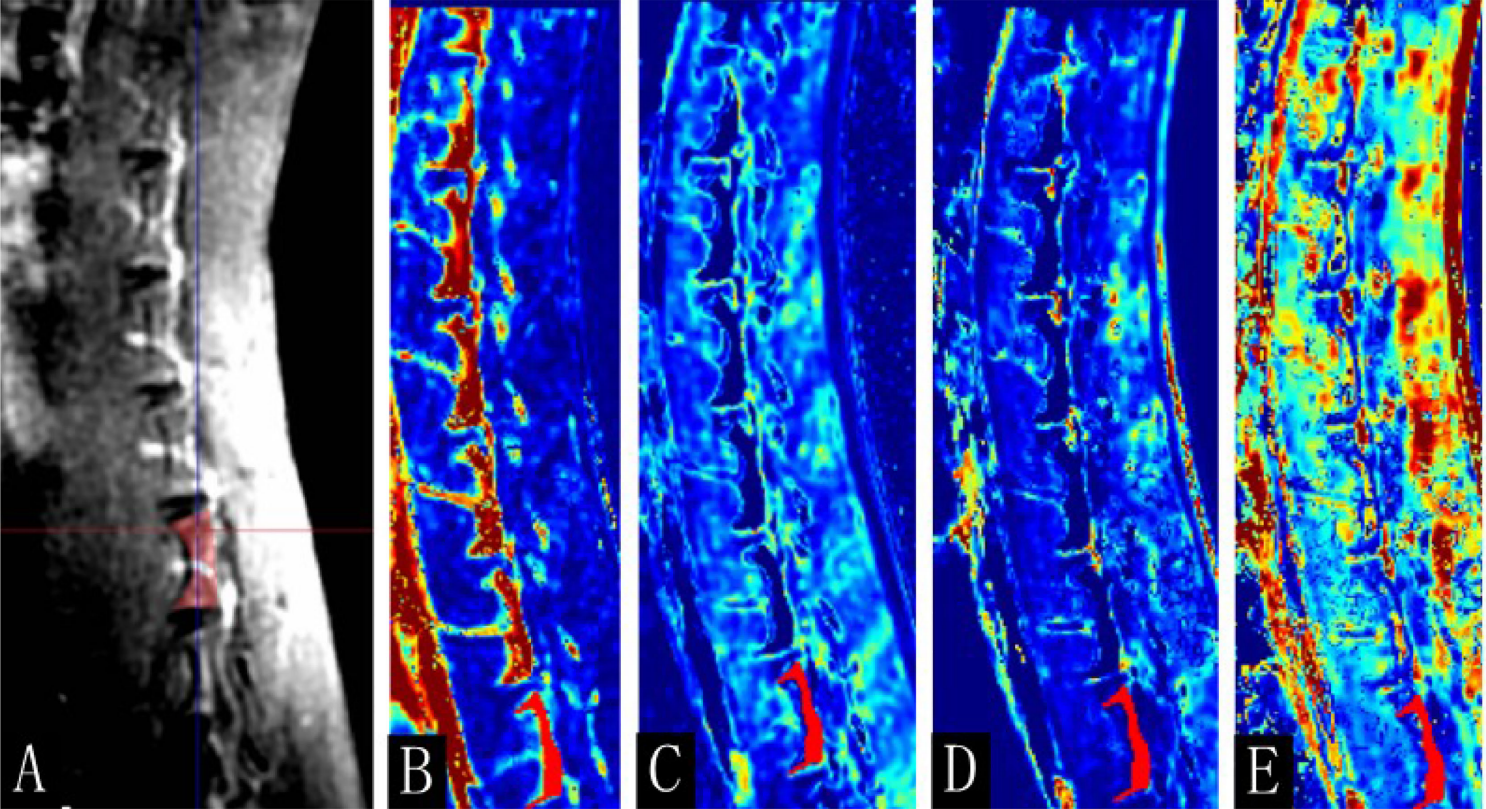
The postprocessing images of dynamic contrast-enhanced magnetic resonance imaging (DCE-MRI). **(A)** Enhanced T_1_-weighted imaging (T_1_WI) map, **(B)** *K^trans^*, **(C)** *K_ep_*, **(D)** *V_e_*, and **(E)** *V_p_* map of the 16th-week normal rabbits. The lumbar vertebra region of interest was manually drawn (red area).

DCE parameters were measured using a quantitative DCE-MRI software package (Omni-Kinetics; GE Healthcare). First, 3D non-rigid registration was performed on all acquired 3D images to reduce respiratory motion artifacts. Subsequently, the liver acquisition volume acceleration images with 9° and 12° of flip angles were introduced for T_1_ mapping calculation. The motion-corrected dynamic images were then used for DCE analysis. During the calculation, the contrast time–concentration curve in the abdominal aorta was selected as the arterial input function of the lumbar spine, and the extended Tofts linear model was used for DCE model fitting. To avoid the intervertebral disc, vertebral venous plexus, and bone island, one region of interest (ROI) was manually outlined at each slice, and all were merged into a 3D volume of interest. Since the image structure of enhanced scan is not as clear as that of sagittal FSE-T_1_WI and FSE-T_2_WI, we used sagittal FSE-T_1_WI and FSE-T_2_WI for comparison during postprocessing to draw the ROI (**Figure 1**). The permeability parameters, including the volume transfer constant (*K^trans^*) between the blood plasma and the extracellular extravascular space, extracellular extravascular volume fraction (*V_e_*), rate constant (*K_ep_* = *K^trans^*/*V_e_*) between the plasma and the extracellular extravascular space volume, and contrast agent plasma volume (*V_p_*), were calculated automatically. The postprocessing images of DCE-MRI are shown in **Figure 2**. High *K^trans^* value indicates high permeability and perfusion volume. *K_ep_* shows the flow between the leakage space and the plasma. In addition, different contrast agents have different *K^trans^* and *V_e_*.

**Figure 3:**
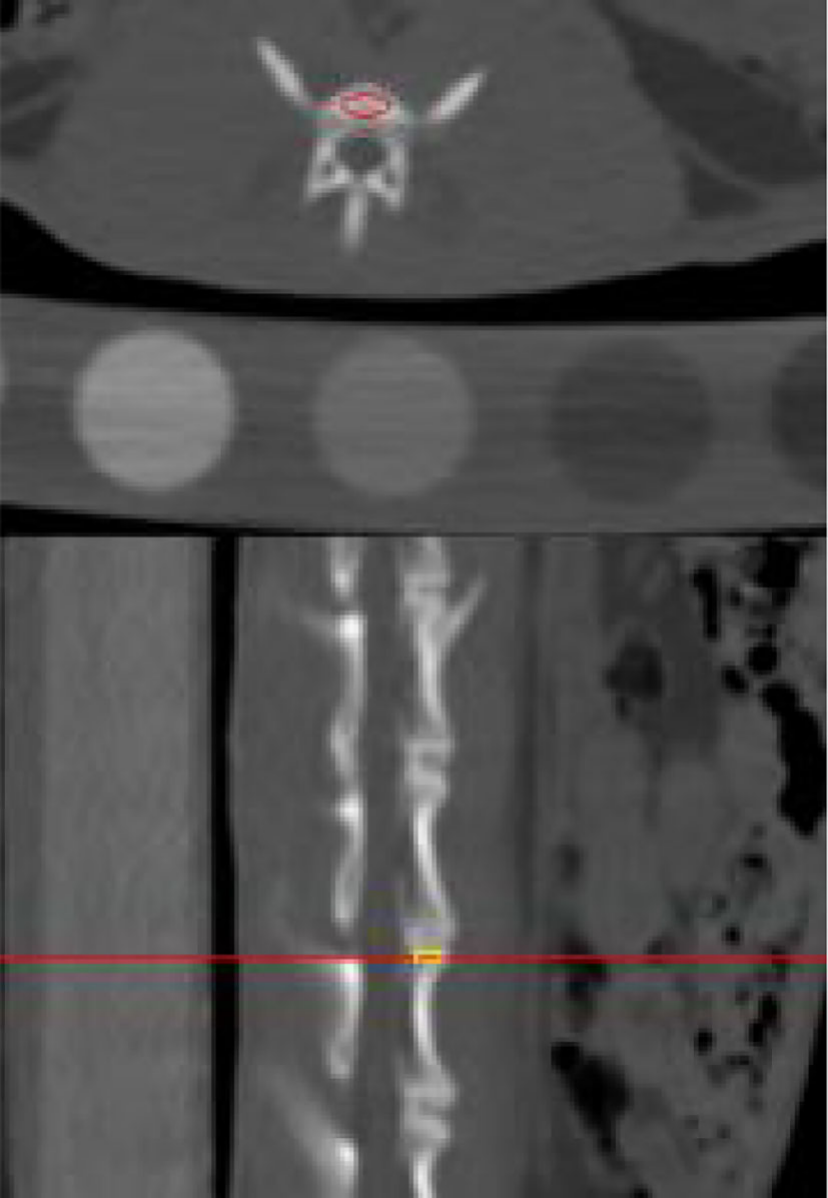
Illustration of the outlined region of interest on postprocessed quantitative computed tomography.

The QCT images were uploaded to the Mindways Pro postprocessing software, and the BMD of the rabbit lumbar 4–7 vertebrae was computed. ROIs were drawn as shown in **Figure 3**.

### Texture Feature Extraction

All images were transferred in digital imaging and communications in medicine format. *K^trans^* map was measured using a quantitative DCE-MRI software package (Omni-Kinetics; GE Healthcare). Sixty-eight texture features including histogram parameters, morphological parameters, gray-level co-occurrence matrix (GLCM) parameters, and gray-level run length matrix parameters in the 3D ROI of *K^trans^* map were extracted for the three repeated measurements. The average of each parameter was also used as the final result.

### Sample Selection and Histologic Analysis

**Figure 4:**
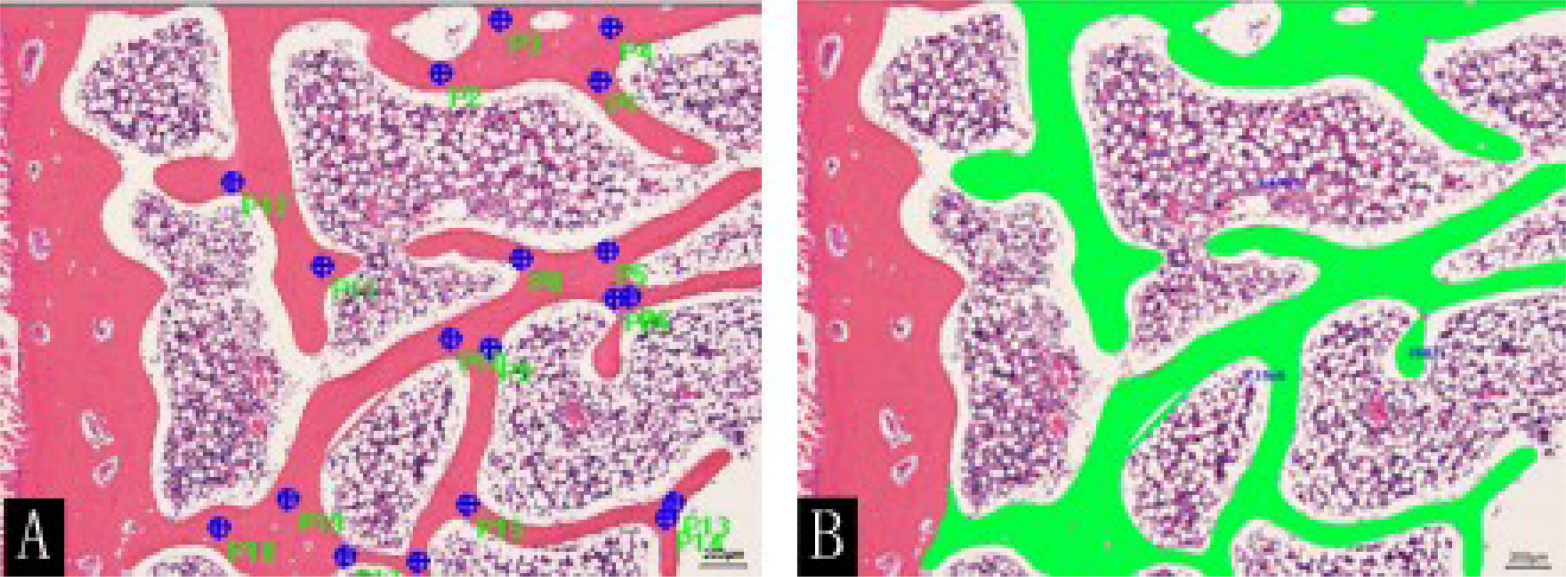
H&E staining (×50) of rabbit lumbar vertebrae in the control group at the 16th week. **(A)** Marks (blue dots) and **(B)** sketches (green color) of the trabecular bone were performed using the Image-Pro Plus software.

After MRI and CT examinations were completed at the 16th week, all rabbits were killed by air embolization. The seventh lumbar vertebral body was fixed with a universal tissue fixative (4% paraformaldehyde buffer solution, neutral pH). After decalcification and paraffin embedding, the largest cross-section of the lumbar vertebral body was selected and made in the thick section of 4 µm. Subsequently, hematoxylin and eosin (H&E) staining and CD34 immunohistochemical staining were performed. Four independent regions with the same area were selected for observation and filming under an optical microscope (OLYMPUS BX51). H&E-stained images were analyzed using Image-Pro Plus 6 (Media Cybernetics, Inc., MD, USA) software to measure structural parameters of the trabecular bone in the sections, including trabecular bone number (Tb.N) and trabecular bone area (Tb.Ar) (**Figure 4**).

### Statistical Analysis

Statistical analyses were performed with SPSS 25.0. Pearson correlation coefficient was used to investigate the correlation between permeability parameters and BMD. Repeated-measures ANOVA was used to analyze the changes in lumbar BMD trend over time in each group and the lumbar marrow texture parameters of diabetic rabbits at different time points. The two-sample t-test or the Mann–Whitney U test was performed to compare the differences of all variables between the diabetic and control groups. Receiver operating characteristic curves were drawn for the parameters with statistical significance. Two-sample t-tests were used to compare the differences in lumbar bone trabecular morphometric parameters between the two groups. Two-sided P < 0.05 was considered statistically significant.

## Results

In the disease progression, four diabetic rabbits died due to bad health conditions, and two normal rabbits died due to poor anesthesia. Thus, 10 rabbits in the diabetic group and eight rabbits in the control group were finally included in the analysis.

**Figure 5:**
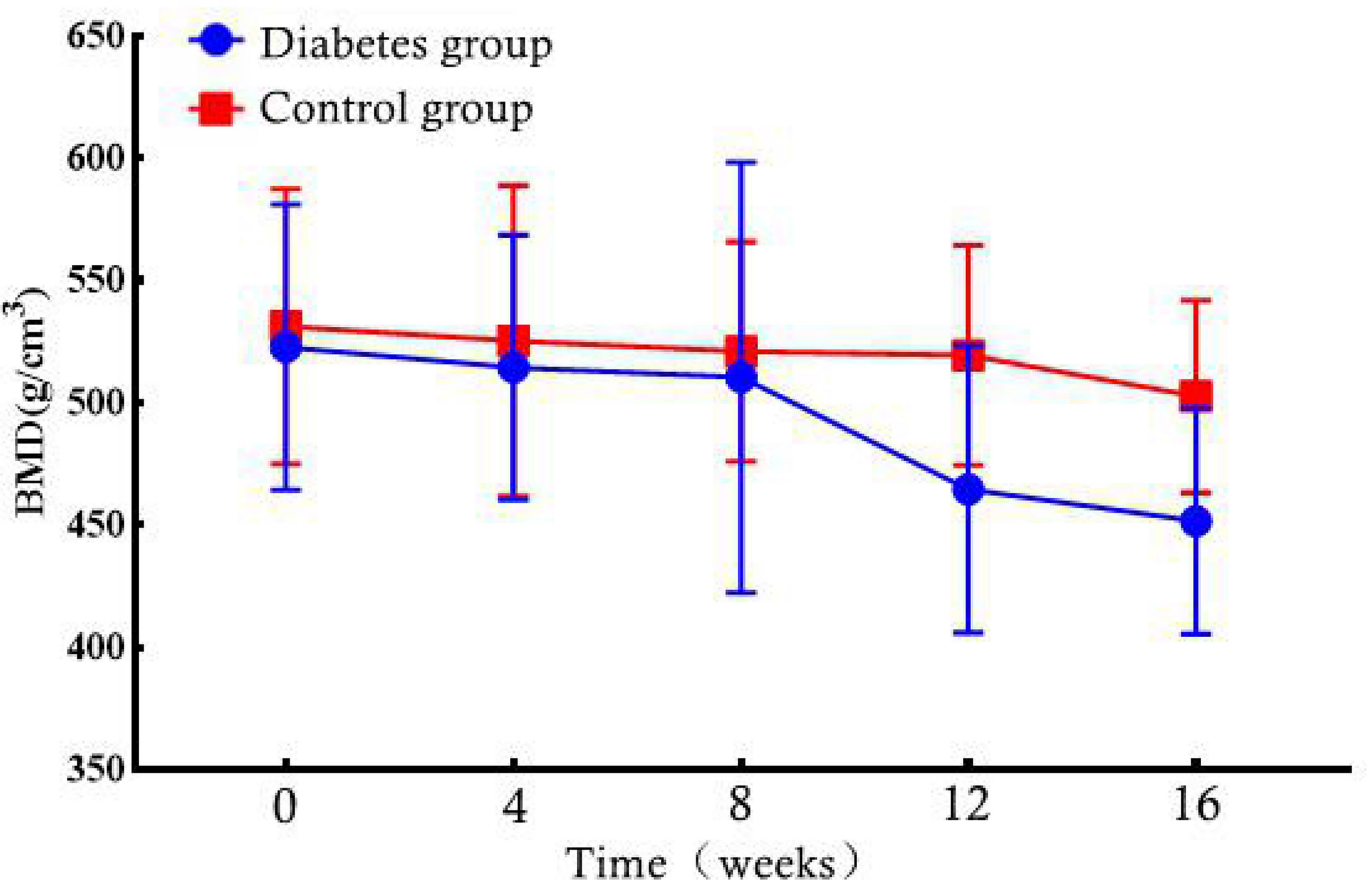
Bone marrow density (BMD) changes in the diabetic and control rabbit lumbar vertebrae over time.

At weeks 0–16, the BMD of the rabbits in the diabetic and normal groups was not statistically significant (P = 0.796, P = 0.75, P = 0.789, P = 0.083, P = 0.055) (**Table 1**). However, when the BMD data of the rabbits in the diabetic group were compared from 0 to 16 weeks, the change in BMD showed an overall downward trend (**Figure 5**).

**Table 1:** Group differences of BMD values in the lumbar vertebrae.

Time	Diabetes group	Control group	*T*	P
0 week	522.81 ± 58.44	531.25 ± 56.29	0.265	0.796
4 weeks	514.45 ± 54.22	525.28 ± 63.48	0.327	0.75
8 weeks	510.39 ± 88.04	520.95 ± 44.87	0.266	0.789
12 weeks	464.63 ± 58.73	519.53 ± 44.97	1.909	0.083
16 weeks	451.69 ± 46.17	502.63 ± 39.35	2.151	0.055

Data are expressed as mean ± standard deviation.

BMD, bone marrow density.

**Figure 6:**
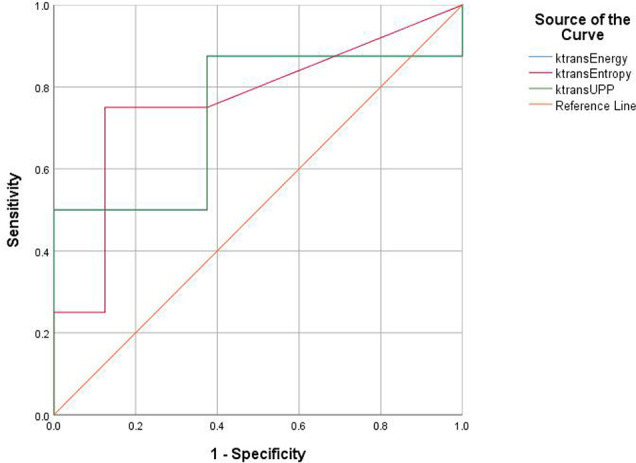
Receiver operating characteristic (ROC) curves demonstrate the diagnostic ability in the differentiation of lumbar bone marrow texture between groups at the 12th week.

**Figure 7:**
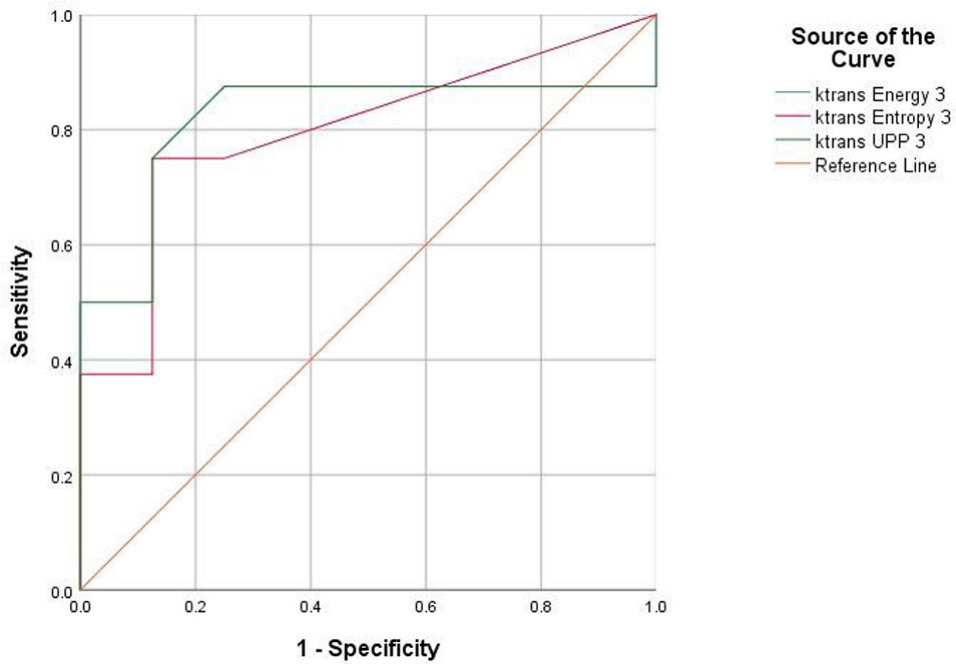
Receiver operating characteristic (ROC) curves demonstrate the diagnostic ability in the differentiation of lumbar bone marrow texture between groups at the eighth week.

For texture analysis, of the 68 parameters extracted from the *K^trans^* map, only entropy, energy, and UPP parameters showed significant differences from week 0 to 16 between the two groups (all P < 0.05) (**Table 2**). Thus, we excluded the remaining 65 parameters, and only the three parameters selected were analyzed using the receiver operating characteristic curves. In the diabetic group, the identification ability at 8–12 weeks was higher than that at 12–16 weeks, and the areas under the curve (AUCs) were 0.734, 0.766, and 0.734, respectively (**Tables 3**, **4** **;** **Figures 6**, **7**).

**Table 2:** Group comparison of *K^trans^*-derived texture parameters at the same time points.

Indicators		Control group			Diabetic group			Z	P-value
		Median	P25	P75	Median	P25	P75		
T0									
	*K^trans^*-Energy	0.500	0.330	1.000	0.330	0.200	1.000	-3.195	0.001
	*K^trans^*-Entropy	0.000	0.000	0.000	0.000	0.000	1.000	-3.605	<0.001
	*K^trans^*-UPP	0.500	0.330	1.000	0.330	0.200	1.000	-3.195	0.001
T1									
	*K^trans^*-Energy	1.000	0.500	1.000	0.500	0.250	1.000	-3.646	<0.001
	*K^trans^*-Entropy	0.000	0.000	0.000	0.000	0.000	0.000	-2.322	0.020
	*K^trans^*-UPP	1.000	0.500	1.000	0.500	0.250	1.000	-3.646	<0.001
T2									
	*K^trans^*-Energy	0.500	0.290	1.000	1.000	0.500	1.000	-2.195	0.028
	*K^trans^*-Entropy	0.000	0.000	0.000	0.000	0.000	0.000	-2.938	0.003
	*K^trans^*-UPP	0.500	0.290	1.000	1.000	0.500	1.000	-2.195	0.028
T3									
	*K^trans^*-Energy	1.000	1.000	1.000	0.500	0.250	1.000	-2.957	0.003
	*K^trans^*-Entropy	0.000	0.000	0.000	0.000	0.000	0.000	-5.474	0.000
	*K^trans^*-UPP	1.000	1.000	1.000	0.500	0.250	1.000	-2.957	0.003
T4									
	*K^trans^*-Energy	1.000	1.000	1.000	1.000	0.330	1.000	-2.448	0.014
	*K^trans^*-Entropy	0.000	0.000	0.000	0.000	0.000	0.000	-3.350	0.001
	*K^trans^*-UPP	1.000	1.000	1.000	1.000	0.330	1.000	-2.448	0.014

Represented that the difference between the normal group and the control group at the same time point was statistically significant (P < 0.05).

**Table 3:** Area under the ROC curve shows the diagnostic ability of lumbar bone marrow texture differentiation by comparing the texture parameters at week 12 when taking week 8 as baseline.

Area under the curve					
Test result variable(s)	Area	Std. error^a^	Asymptotic sig.^b^	Asymptotic 95% confidence interval	
				Lower bound	Upper bound
*K^trans^*-Energy	0.734	0.136	0.115	0.469	1.000
*K^trans^*-Entropy	0.766	0.128	0.074	0.516	1.000
*K^trans^*-UPP	0.734	0.136	0.115	0.469	1.000

Test result variable(s): K^trans^-Entropy has at least one tie between the positive actual state group and the negative actual state group. Statistics may be biased.

a Under the nonparametric assumption.

b Null hypothesis: true area = 0.5.

ROC, receiver operating characteristic.

**Table 4:** Area under the ROC curve shows the diagnostic ability of the texture parameters at week 16 compared with week 12 at baseline.

Area under the curve					
Test result variable(s)	Area	Std. error^a^	Asymptotic Sig. ^b^	Asymptotic 95% confidence interval	
				Lower bound	Upper bound
*K^trans^*-Energy	0.508	0.153	0.958	0.208	0.808
*K^trans^*-Entropy	0.594	0.148	0.529	0.304	0.884
*K^trans^*-UPP	0.508	0.153	0.958	0.208	0.808

Test result variable(s): K^trans^-Energy, K^trans^-Entropy, and K^trans^-UPP have at least one tie between the positive actual state group and the negative actual state group. Statistics may be biased.

a Under the nonparametric assumption.

b Null hypothesis: true area = 0.5.

ROC, receiver operating characteristic; UPP, uniformized positive pixel.

Pearson correlation coefficient showed that BMD was correlated with *K^trans^*, *K_ep_*, and *V_e_* (r = 0.134, 0.299, and -0.252; P ≤ 0.001) but not correlated with *V_p_* (r = -0.038, P = 0.373) (**Table 5**).

**Table 5:** Pearson correlation coefficients of BMMP parameters and BMD.

Correlation with BMD	r	P
*K^trans^*	0.134	0.001
*K_ep_*	0.299	<0.001
*V_e_*	-0.252	<0.001
*V_p_*	-0.038	0.373

BMMP, bone marrow microvascular permeability; BMD, bone marrow density.

**Figure 8:**
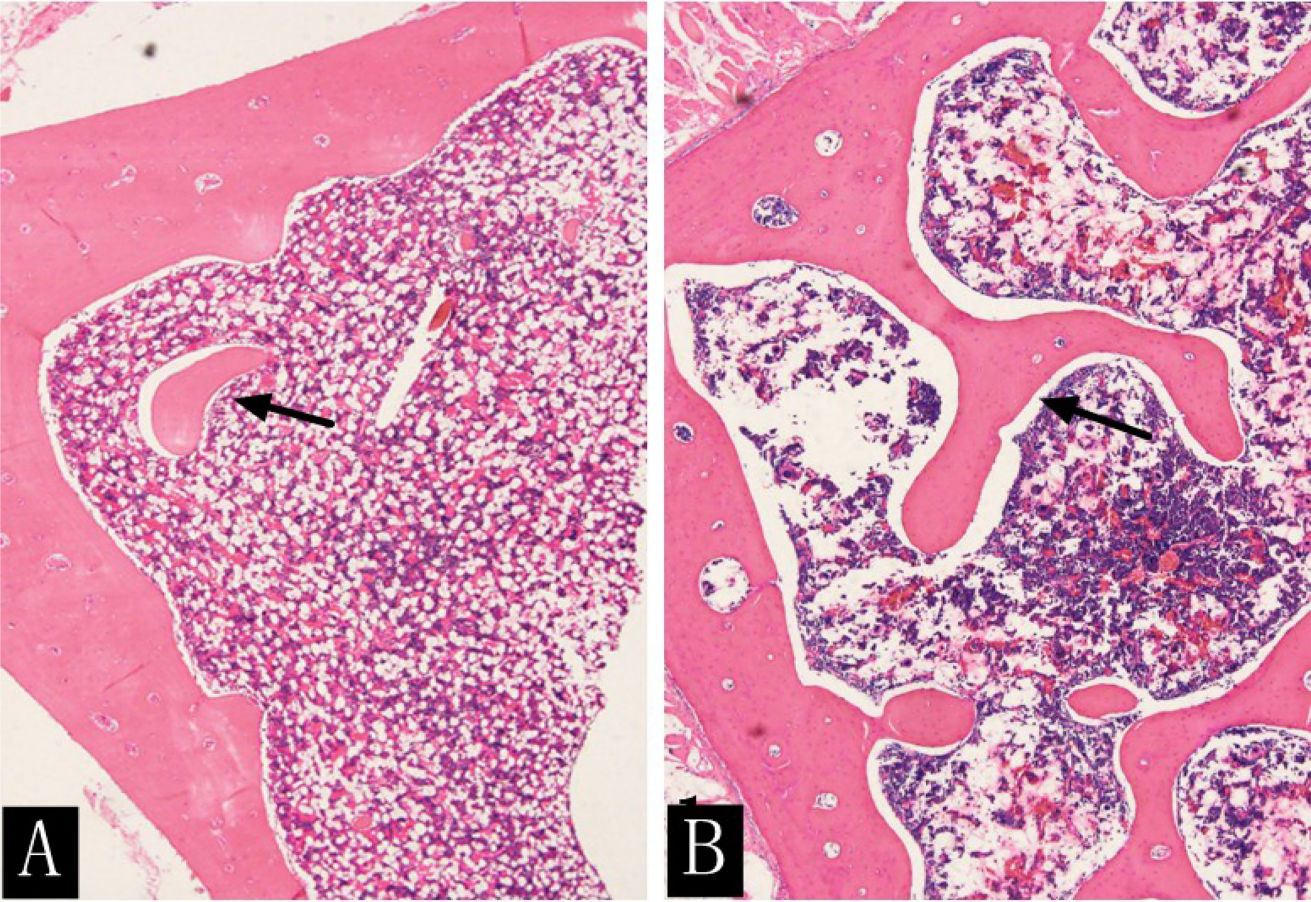
H&E staining (×400) of rabbit lumbar vertebrae in the diabetic group **(A)** and the control group **(B)** at the 16th week. The rabbit lumbar trabecular bone was more densely distributed and had a larger area in the control group than in the diabetic group (arrow).

**Figure 9:**
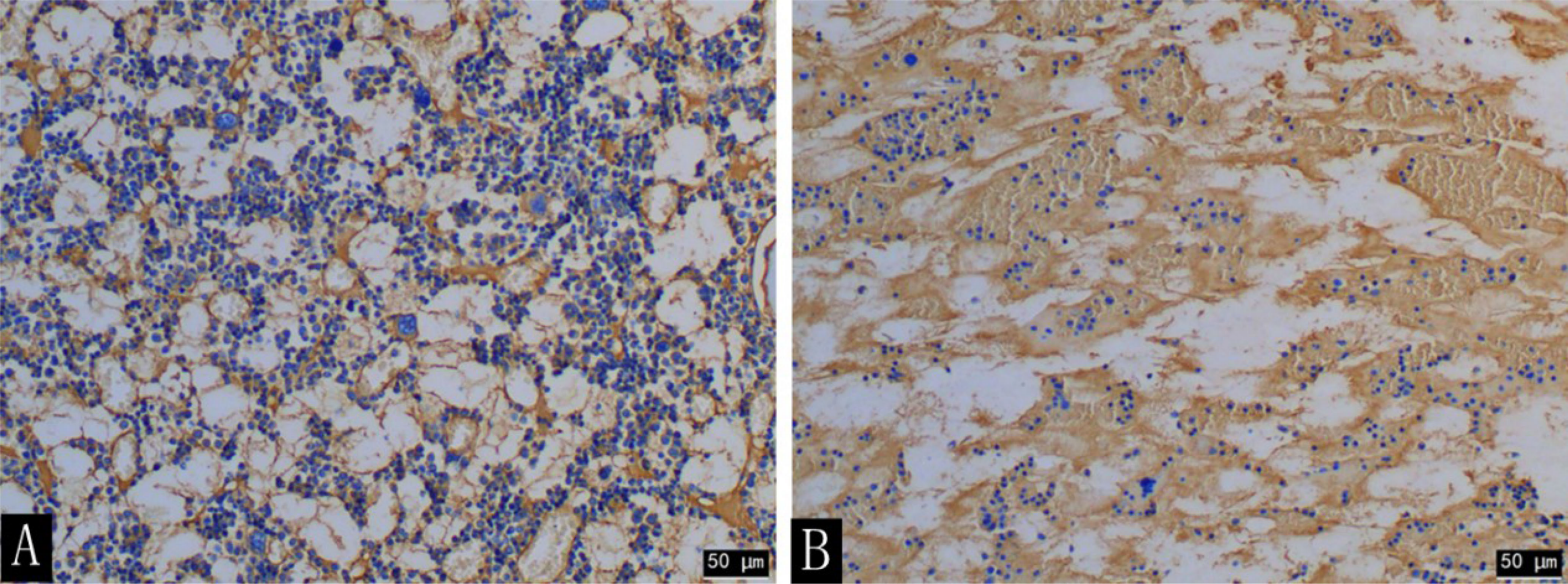
CD34 immunohistochemical staining (×200) of the rabbit lumbar bone marrow in the diabetic group **(A)** and the control group **(B)** at the 16th week. The number of cells in the lumbar bone marrow was significantly increased in the diabetic group compared with that in the control group.

At the time of sacrifice (after 16 weeks), the H&E staining results of the lumbar vertebrae in the diabetic group showed that the decreased number of trabecular bone and smaller area was observed with the naked eye and statistically different from that of the control group (t = 12.472; t = 4.961; P < 0.001) (**Figure 8**, **Table 6**). The immunohistochemistry of bone marrow CD34 showed that the number of cells in the bone marrow increased significantly and more widespread, indicating increased heterogeneity of images (**Figures 9**).

**Table 6:** Comparison of the number of trabecular bone and trabecular bone area in the diabetic and control groups at the 16th week.

16th week	Diabetes group	Control group	*Z*	P
Tb.N				
	6.00 (4.00–11.00)	21.50 (17.50–26.00)	3.863	<0.001
Tb.Ar				
	574,017.64 (279,256.13–847,322.88)	1,825,414.82 (1,307,484.69–2,063,523.82)	3.627	<0.001

Data are expressed as mean (range).

Tb.N, trabecular bone number; Tb.Ar, trabecular bone area.

## Discussion

This study used both DCE-MRI and QCT as well as texture analysis to evaluate the changes in bone marrow microcirculation and BMD in the alloxan-induced diabetic rabbit model. The results showed that DCE-MRI permeability parameters of the lumbar spinal marrow were correlated with BMD. No statistically significant changes were found in BMD in the diabetic group during the disease progression. However, the trabecular bone histometry parameters were statistically different between the baseline and the 16th week and between groups except for baseline. The histogram and GLCM of *K^trans^* map showed viability of identifying early microstructural changes in diabetic bone marrows.

Oikawa et al. (18) initially showed that the femoral and tibial bone marrow microvascular diseases in T1D rats occur; osteopenia, bone marrow fat cell accumulation, increment of microvascular permeability, and reduction of blood flow volume, arteriole, capillary network, and bone marrow sinusoids were observed. Hu et al (11) found significantly increased microvascular permeability and fat content, decreased microvessel density of bone marrow, and destroyed microenvironment of bone marrow osteoblasts in diabetic rabbit lumbar vertebra after 12 weeks. Our data showed that the microvascular permeability of diabetic rabbit bone marrow decreased during the experiment. A clear correlation was noted between changes in bone marrow microvascular permeability and BMD at early-stage diabetes. Diabetic bone metabolism disorders mainly include osteopenia and osteoporosis. Diabetes with bone marrow microvascular disease can aggravate bone metabolic disorders, and diabetes with a primary osteoporosis disease can exacerbate the condition of osteoporosis in patients (9). Bone marrow microangiopathy is an important indicator of risk, prevention, and treatment for bone diseases. Starting a long-term prospective research before the progression of microvascular disease and performing large-scale epidemiological studies are necessary to better understand the relationship between bone marrow microangiopathy and BMD changes and fracture risks.

The pathophysiological mechanisms of early diabetic bone marrow microangiopathy in either type 1 or 2 diabetes (T2D) have not been elucidated (9, 18). The QCT results in our study showed that the BMD of the lumbar vertebra in the diabetic group had a downward trend without significant differences to that of the control group, in accordance with the results obtained by Register et al. (19). At present, no T1D-related QCT studies have been performed, and only type II diabetes-related QCT studies are used as a reference for comparison at the 16th week. The H&E staining results showed that the number of trabecular bone and trabecular bone area was significantly smaller in the diabetic group than that in the control group. No correlation was found between BMD and trabecular bone parameters. We speculated that identification of changes in lumbar vertebra BMD by using QCT only partially reflected the histological changes in trabecular morphometry. Since QCT in T1D is rarely applied and a study has shown that the high-resolution peripheral QCT parameters are not different between type 1 and 2 diabetes patients in the adjusted analyses except for an increased stiffness at the tibia in T2D patients (20), it is only possible to explain our QCT results based on previous T2D research. The microfinite element analysis showed that bone strength in T2D patients is associated with increased cortical porosity in the distal radius compared with that in healthy controls (21, 22). In addition, cortical osteoporosis and trabecular heterogeneity are more pronounced in T2D patients with fractures compared with T2D patients without fractures (23). Higher cortical and periosteal cortical voids in T2D patients with fractures compared with those without fractures suggest that cortical subchambers may be more sensitive to the T2D-induced toxicity and reflect the extent of microvascular disease lesions (24). In our study, the delineated ROI on QCT images without including the cortical area when measuring BMD may explain the insignificantly declined BMD. Future research should focus on evaluating the structural determinants (microstructure, material properties) of bone fragility to assess the accuracy and reproducibility of QCT for measuring lumbar BMD in diabetic rabbit models.

In our study, some texture parameters based on the *K^trans^* map showed differences between the diabetic and control groups. “Entropy,” “UPP,” and “energy” at eighth, 12th, and 16th week were significantly different between the two groups. Moreover, the diagnostic efficiency of texture parameters at 8–16 weeks was higher than that at 12–16 weeks. The AUC of entropy was the largest among all texture parameters, indicating that entropy is more effective in identifying early microstructural changes in diabetic bone marrow.

Entropy has been used for computer-aided evaluation of bone regeneration process and structural complexity (e.g., the formation of mature trabecular bone) (25). Mookiah et al. (26) showed that some GLCM texture parameters (energy, entropy, uniformity) acquired from routine-enhanced multidetector CT and analyzed with a single vector machine could be applied to screen opportunistic osteoporosis. Higher GLCM entropy in the diabetic group than that in the control group indicated that the texture distribution was more complicated.

The microscope-scale results of texture analysis and histopathology were different, but a certain relationship existed between them. MacKay et al. (15) reported that texture features of the high-spatial resolution coronal T_1_-weighted MRI images (e.g., histogram variance) acquired from 10 patients with knee arthritis aged 57–84 years are significantly correlated with the histological parameters of the subchondral bone tissue of the tibial plateau during total knee arthroplasty. In our study, CD34 immunohistochemical pathology showed that the number of bone marrow cells was higher, and the distribution range was wider in the lumbar vertebra of the diabetic group than that of the control group, leading to elevated regional variation and tissue complexity. This was reflected by increased entropy. In addition, lower BMD in the diabetic rabbits than in the control group could result in the distinct distribution of contrast agents in the bone marrow, which was also the possible reason for the increased entropy of *K^trans^* map in this study.

In addition, we also found that the “energy” value in the texture parameters had a higher ability to identify the changes in bone microstructure in the diabetic group. Shu et al. (27) showed that the statistical parameter “energy” extracted from the gray intensity distribution is able to distinguish the femoral cortical bone development in two different diet groups.

This study has several limitations. First, the experimental objects were T1D-like rabbits. The incidence of T1D increased with age. The highest incidence occurs in the 10–14-year-old population (1). The New Zealand white rabbits used in the study were 8–9 weeks old, equivalent to 10 human years. The experiment lasted 16 weeks, and the age of the rabbits was about that of an 18-year-old human at the end of the experiment. Our experimental results only indicated no correlation between the changes in bone marrow microvascular permeability and BMD at the early stage of diabetes; thus, variation of more complete time series between the two factors remains to be further studied. Second, this experiment failed to observe the results of the trabecular bone specimens in the entire process. Further improvement in the subsequent research is warranted. Finally, our experimental sample size was small, and the AUCs of the texture parameters with statistically significant differences between the diabetic and control groups were between 0.5 and 0.8, indicating that the diagnostic efficiency is moderate. Detailed information on the changes in texture characteristics of microvascular disease should be further verified by large sample experiments and radiomic methods.

In summary, no correlation was found between the changes in microvascular permeability parameters of lumbar spine bone marrow and bone density and trabecular morphometric parameters at the early stage of diabetes model induced by alloxan. The bone marrow BMD changes measured using QCT occurred later than those measured using trabecular morphometrics. The texture analysis based on the DCE-MRI *K^trans^* map could identify bone marrow microstructure changes at the early stage of diabetes.

## Data Availability Statement

The raw data supporting the conclusions of this article will be made available by the authors without undue reservation.

## Ethics Statement

The animal study was reviewed and approved by the ethics committee of Wuhan University.

## Author Contributions

YZ and PC incubated and designed the experiments. PC, BL, QY, and LY conducted the research. PC, HL, and WL analyzed and interpreted the data. PC drafted this article. YZ made a critical review of the intellectual content of the article. LW, LL, LH, DX, and CL provided administrative, technical, or material support. All authors contributed to the article and approved the submitted version.

## Funding

This study was funded by the National Natural Science Foundation of China (nos. 81871332).

## Conflict of Interest

The authors declare that the research was conducted in the absence of any commercial or financial relationships that could be construed as a potential conflict of interest.

## Publisher’s Note

All claims expressed in this article are solely those of the authors and do not necessarily represent those of their affiliated organizations, or those of the publisher, the editors and the reviewers. Any product that may be evaluated in this article, or claim that may be made by its manufacturer, is not guaranteed or endorsed by the publisher.
